# The effect of multiple martensitic transformations on diffusion of Fe and Ni atoms in Fe-31.7%Ni-0.06%C alloy

**DOI:** 10.1186/1556-276X-9-322

**Published:** 2014-06-27

**Authors:** Vitaliy E Danilchenko, Vladimir F Mazanko, Viktor E Iakovlev

**Affiliations:** 1 G.V. Kurdyumov Institute for Metal Physics, NAS of Ukraine, Vernadsky Blvd. 36, Kyiv 03142, Ukraine

**Keywords:** Diffusion, Radioisotope, Nanofragment, Dislocations density, Martensitic transformation

## Abstract

Diffusion characteristics of iron and nickel atoms were investigated using radioactive isotopes method in phase-hardened metastable iron-nickel Fe-31.7%Ni-0.06%C alloy with nanofragmented structure. It has been found that diffusion mobility of nickel and iron atoms in reverted austenite of Fe-31.7%Ni-0.06%C alloy significantly increases as the result of multiple *γ*-α-γ martensitic transformations. The diffusion coefficients of nickel and iron in the austenite at 400°C corresponded to the stationary diffusion coefficients at the temperatures above 900°C. The revealed diffusion acceleration at low temperatures is caused by high-density dislocations and additional low-angle subboundaries of disoriented nanofragments of reverted austenite and deformation twin subboundaries formed during multiple γ-α-γ cycles.

## Background

Diffusion in metallic materials plays a significant role in grain boundary processes and, hence, helps forming the whole spectra of physical and mechanical properties of such materials as well as affects performance of metallic materials' products. By changing diffusion parameters one way or another, we can purposefully operate the performance properties of metals and alloys. A variety of ways have been elaborated to affect the diffusion mobility of the atoms in metallic materials. The primary ones include diffusion annealing at different temperatures [1], thermal cycling [2,3], plastic deformation [4-6], high-energy treatment (plasma, laser emission, electric spark, etc.) [1], and phase transformations of various types [7-14].

Martensitic transformations are the ones that most significantly affect the diffusion properties of interstitials and substitution atoms since during their course in the initial phase of metastable alloys, the dislocation density increases considerably and additional subboundaries are formed. These changes and the formation of a specific structural state of an alloy are able to increase significantly (by orders) the diffusion mobility of atoms at temperatures below 0.5 of melting point. In iron-nickel alloys, γ-α-γ transformations are obtained with face-centered cubic (f.c.c.)-body-centered cubic (b.c.c.)-f.c.c. structure rebuilding, whereas in ferromanganese alloys one gets γ-ϵ-γ and γ-ϵ′-γ transformations with f.c.c.-hexagonal close-packed (h.c.p.)-f.c.c. and f.c.c.-18-layer rhombic (18R)-f.c.c. structure rebuilding [15], respectively. In our study, dislocation density in the reverted austenite increased by more than three orders as the result of multiple γ-α-γ transformations. After γ-ϵ-γ transformations dislocation density increased not more than by one order, and after γ-ϵ′-γ transformations, it remained practically unchanged. We associate this regularity with different volume effects of direct martensitic transformation. Such γ-α, γ-ϵ, and γ-ϵ′ transformations are accompanied by a specific volume increase, namely, by 3.4%, 1.75%, and 0.5%, respectively. In the ferromanganese-reverted austenite, multiple γ-ϵ-γ transformations caused the accumulation of random packing defects, and γ-ϵ′-γ transformations remained at practically same numbers. In the case of multiple γ-α-γ transformations, under the generation of new dislocations during subsequent cycles and their accumulation and interaction, additional subboundaries arose, for example, through forming the walls of one-sign dislocations. Due to this process, highly dispersed disoriented fragments of reverted austenite were formed. The accumulation of packaging defects in ferromanganese alloys does not lead to the forming of additional subboundaries and fragmented structural elements. For these reasons, the greatest influence on diffusion characteristics of alloying elements among different types of martensitic transformations in alloys based on iron was the γ-α-γ transformations generating significant numbers of dislocations in the initial phase.

We are not aware of any investigations concerning how γ-α-γ transformations influence on the diffusion properties of substitution atoms. As the result of γ-α-γ transformations, crystal structure has been formed having a system of defects (dislocations, low-angle subboundaries, deformation twins), different from the ones received in case of γ-ϵ-γ transformations (dislocations, packaging defects). Different structure defects may have different influence on diffusion processes. In our work, we studied the influence of defects in crystal structure, which have been formed as the result of γ-α-γ transformations, on the diffusion properties of nickel and iron atoms in Fe-31.7%Ni-0.06 %C alloy.

## Methods

Fe-31.7%Ni-0.06%C alloy was in austenite state at room temperature. The direct, γ-α transformation in the alloy, occurred as the result of cooling in liquid nitrogen, and the reverse, α-γ one, during consequent heating in a salt bath at the temperature of 400°C. In our experiments, the heating rate of hardened samples under the inverse transformation was 80°/sec. To avoid relaxation processes in the reverted austenite, we prevented overheating above the temperature at the final point of inverse transformation. Temperature range of the direct and the reverse martensitic transformations was defined by a differential magnetometer. The magnetic field shown by the magnetometer was 10 kOe; the temperature was measured in the range of -196°C to 500°C, the amount of martensitic phase was measured with the accuracy of 0.5%. The temperature points of the investigated alloy were *M*_*s*_ = -60°C, *M*_*f*_ = -160°C, *A*_*s*_ = 290°C, and *A*_*f*_ = 400°C. The measurement accuracy of the diffusion coefficient was 20%. Phase analysis was performed on automatic X-ray diffractometer DRON-3 (Moscow, USSR). Electron microscopic research was performed using microscope PREM-200 (Moscow, USSR).

A layer with radioactive isotope ^63^Ni or a mixture of isotopes ^55,59^Fe was deposited on one of the austenitic alloy surfaces. The thickness of the isotope was less than 0.5 μm and β activity was (5 × 10^3^ ± 50) pulses/min. Concentration distribution of nickel and iron (in different samples) in depth after the multiple martensitic γ-α-γ transformations and diffusion annealing at temperatures 400°C was obtained using photographic method, with exposing the film to X-rays in a vacuum for 30 days. The employed photographic method is based on the interaction of radiation with a photosensitive emulsion film. This method is not a destructive one. After exposing and developing, the blackenings on the films were analyzed using computer-analyzer photometer MF-4.

To calculate the diffusion coefficient, we used the following formula (from Fick's first law) [16]:

$$D=-\left(\frac{1}{4t\times \mathrm{tg}\left(\alpha \right)}\right),$$

where *tg* (*α*) was determined from the graph:

$$f\left(x_{n}^{2}\right)=\mathrm{lg}\left(\frac{\partial I_{n}}{\partial x_{n}}\right),$$

where *t* is the diffusion annealing time, *x* is the penetration depth of the isotope into the sample, and *I* is the intensity of the isotope at a certain depth.

## Results and discussion

After cooling in liquid nitrogen, the alloy contained 85% of martensite phase. Multiple γ-α-γ transformations by rapid cooling under the direct γ-α transformation and rapid heating under the reverse α-γ transformation did not lead to significant stabilization of the reverted austenite towards next γ-α transformation. So, after ten cycles of γ-α-γ transformations, the amount of martensite phase, when cooled in liquid nitrogen, decreased by 5% to 7%, whereas after 50 cycles, by only 8% to 10%. The slight decrease of the martensite phase after repeated temperature cycling made it possible to achieve a high degree of phase hardening rate of the reverted austenite under γ-α-γ transformations and generate highly dispersed disoriented fragments of γ-phase.

**Figure 1:**
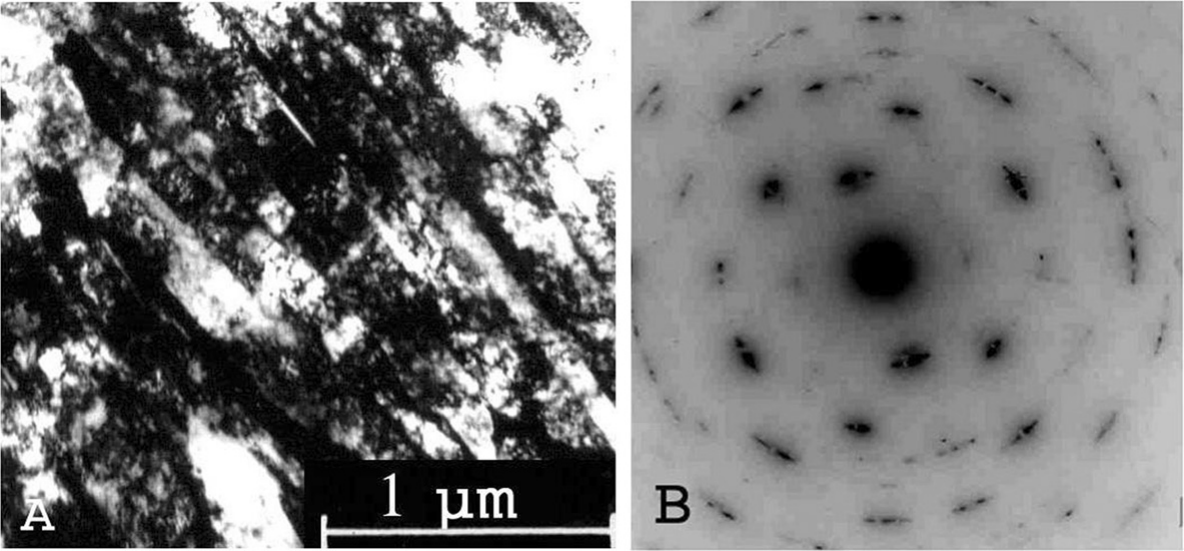
**Microstructure (A) and electron diffraction pattern of reverted austenite (B) after 50 γ-α-γ transitions.** ×20,000.

Electron microscope research have shown [17] that, after the first γ-α-γ transformation, dislocation density in reverted austenite increases by three orders and reaches the value of 5 × 10^11^ cm^-2^, which fully agrees with [18]. Repeated γ-α-γ transformations slightly increase dislocation density achieved after the first cycle. In reverted austenite, there appear fragments with their size decreased, depending on the increasing number of γ-α-γ transformations, i.e., with the increase of phase hardening degree (Figure 1A). Simultaneously, we observed an increase of azimuthal reflections' blurring of austenite at an early stage of thermal cycling (3 to 5 cycles) and subsequent reflections' partitioning on several components already after 5 to 8 thermocycles. The azimuthal blurring indicated the formation of additional subboundaries with subsequent fragments formation. As the result of multiplied thermocycles, the fragment size reached a nanoscale level - a significant volume fraction of the fragments had a size range of 80 to 150 nm. Grain size was determined from electron micrographs. Further fragmentation rate significantly slowed down with increased number of thermocycles, and it was impossible to achieve a significant reduction of the minimum size of the fragments.The electron diffraction pattern of reverted austenite after 50 γ-α-γ transformations shows that all reflections are divided into several components (Figure 1B). This means that during thermocycling, a number of high angle fragments' boundaries were formed, which thus became already dispersed grains in γ-phase. It is important to note that the formation of grains with high-angle boundaries was already present in the first 3 to 10 cycles of thermocycling, and under further thermocycling, this process has not gained significant development.The presence of substructure elements with straight boundaries (Figure 1) proved the formation of deformed twins of phase-hardened austenite, and they grew with the increase of phase-hardening degree due to accumulation of internal stresses in the reverted γ-phase. Thus, as the result of multiple cycles of γ-α-γ transformations in the reverted austenite in iron-nickel alloy, the dislocations density increased by three orders, nanoscale level fragments (nanofragmentation) with additional small-angle subboundaries were formed, a quantity of dispersed grains having high-angle boundaries increased, and deformation twins came into existence.

The phase-hardened alloy was annealed at temperatures of 400°C for 6 h. As the result of phase hardening, the microhardness of the surface layer of the alloy significantly increased. In the initial austenite state (prior to martensitic transformations), microhardness was equal to 1,159 MPa, and after 10 and 50 γ-α-γ cycles, it increased up to 1,550 and 1,776 MPa, respectively. This pointed to the fact of an increasing degree of reverted austenite strengthening under the consistent reiteration of γ-α-γ cycles.

**Figure 2:**
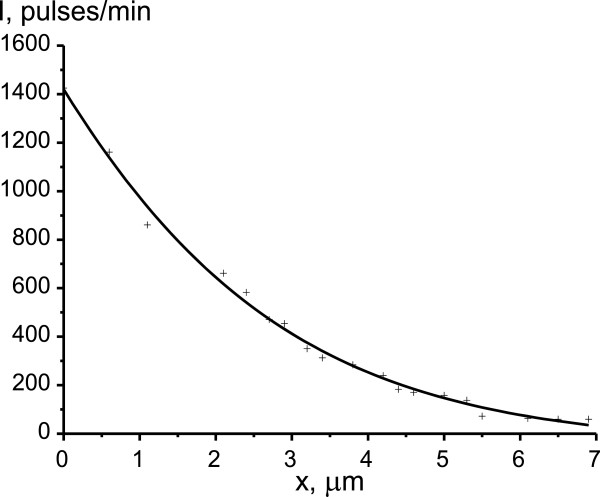
**Concentration distribution of the **^**63**^**Ni radioisotope in reverted austenite.**

**Figure 3:**
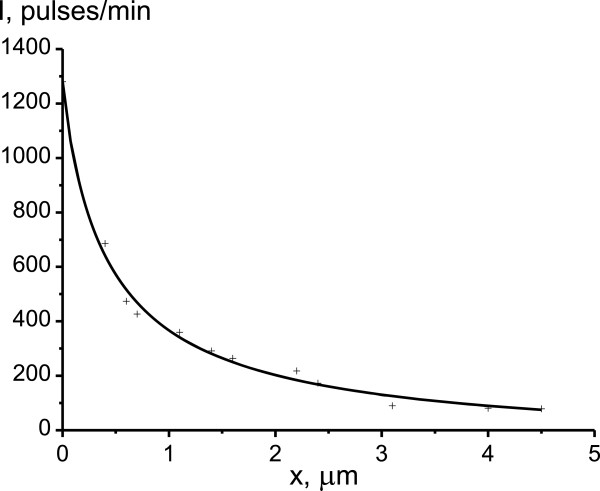
**Concentration distribution of the **^**55,59**^**Fe radioisotopes in reverted austenite.**

Photosensitive film blackening curves that characterize the concentration distribution of the isotopes ^63^Ni and ^55,59^Fe are shown in Figures 2 and 3. Obtained from semilogarithmic curve of the β activity dependence on penetration depth of radioisotopes, the diffusion coefficients of nickel and iron were equal to *D*^Ni^ = 1.14 × 10^-12^ and *D*^Fe^ = 0.86 × 10^-12^ cm^2^/s, respectively. It is evident that the diffusion mobility of nickel in the studied alloy is higher than that of iron. The *D*^Ni^/*D*^Fe^ ratio is equal to about 1.3. This result is qualitatively consistent with the data on the diffusion of nickel and iron in iron-nickel alloy obtained under conditions of stationary isothermal annealing at temperatures higher than 900°C [19]. Such high values of *D*^Ni^ and *D*^Fe^ for relatively low temperature of 400°C are associated with high density of dislocations and high length of additional boundaries and subboundaries between the structural elements that were formed as the result of multiple γ-α-γ transformations.

It was shown, both experimentally and theoretically [6,20], that the dislocations increase diffusion penetration in solids. The contribution of dislocations to the total diffusion flow must be considered mainly at temperatures below 0.5 of melting point. Analysis of experimental data by different authors shows that diffusion coefficients of substitution atoms and interstitials in this temperature range significantly increase depending on dislocation density and grain boundaries length. Diffusion acceleration in defects area of crystal structure is described in [6,8,10,13,20]. The dislocations are those that make the main contribution to the diffusion flow at low temperatures after the first cycle of martensitic transformations. Additional subboundaries give their contributions to the diffusion flow after 20 to 30 cycles of γ-α-γ transformations. Diffusion coefficients were too high - more than 10^3^ times higher compared to the values obtained by extrapolation to high temperature data at temperatures below 0.5 of melting point. Data in this work also show high diffusion transparency of fragments' subboundaries of nanoscale level (nanofragments) due to dislocation nature of small-angle boundaries. We might probably determine the effect of dislocations and additional subboundaries in reverted f.c.c. austenite and b.c.c. martensite onto the total diffusion flow if we studied alloy diffusion characteristics after different numbers of γ-α-γ cycles. It is known that dislocation density increases by three orders after the first γ-α-γ transformation. With increased number of such cycles, dislocation density remains almost unchanged although the total length of additional subboundaries significantly increases [17,18]. The up-to-date ability to create ultrafine and nanocrystalline structures of metallic materials opens new prospects for further intensification methods of chemical and thermal treatment (carburizing, nitriding, metallization) due to a significant acceleration of diffusion. Thus, it follows from this work that temperature of the surface metallization of metastable iron-nickel alloy can be reduced by several hundred degrees. Previously, it has been found [6] that anomalies of grain-boundary diffusion occur in new classes of nanostructured materials created by means of severe plastic deformations. This means that diffusion coefficients increase by several orders and diffusion energy activation is reduced almost by half. Grain-boundary diffusion plays a significant role in the formation of structure-sensitive properties. The authors of [6] believe that this type of diffusion determines significantly the course of diffusion-controlled processes such as recrystallization, high-temperature plastic deformation, superplastic fluidity, temperature-dependent internal friction, and grain-boundary deformation under conditions of fatigue. Diffusion mobility increase of substitution atoms in reverted austenite as the result of multiple martensitic transformation is comparable with the one which occurs as the result of severe plastic deformation.

## Conclusions

As the result of multiple martensitic γ-α-γ transformations, diffusion mobility of nickel and iron atoms in reverted austenite of Fe-31.7%Ni-0.06%C alloy is significantly increased. The diffusion coefficients increased, and at the temperature of 400°C, they corresponded to stationary diffusion coefficients at 900°C. Two factors influenced the diffusion acceleration: a three-order increase of the dislocation density that reached the value of 5 × 10^11^ cm^-2^, and additional low-angle subboundaries of disoriented nanofragments with deformation twins subboundaries formed as the result of γ-α-γ cycles. Low-temperature diffusion anomaly in iron-nickel alloy after multiple γ-α-γ transformations was similar to that described in literature on diffusion abnormalities in nanocrystalline materials obtained by gas condensation method, electrodeposition method, and intensive plastic deformation.

The established regularity of diffusion acceleration of substitution atoms under multiple γ-α-γ martensitic transformations can be used to intensify treatment modes of chemical and thermal treatment, in particular for surface saturation of iron alloys with metals.

## Competing interests

The authors declare that they have no competing interests.

## Authors' contributions

VM carried out the radioisotope investigations and participated in drafting the manuscript. VD has formulated the main idea of investigation and is responsible for all aspects of the work. He also revised critically the manuscript for important intellectual content. VI has prepared all the alloys and specimens, has taken part in acquisition and interpretation of data, and has been involved in drafting the manuscript. All authors have read and approved the final manuscript.
